# Optimization of the reverse transcriptase polymerase chain reaction for the detection of circulating prostate cells

**DOI:** 10.1054/bjoc.2000.1417

**Published:** 2000-10

**Authors:** I G McIntyre, K Spreckley, R B Clarke, E Anderson, N W Clarke, N J R George

**Affiliations:** Department of Urology, Withington Hospital, Manchester, M21 1AH; Clinical Research Department, Christie Hospital, Manchester, M20 4BX; Department of Urology, Hope Hospital, Stott Lane, Salford, M6 8HD, UK

**Keywords:** prostate cancer, reverse transcriptase polymerase chain reaction, micrometastasis

## Abstract

The reverse transcriptase polymerase chain reaction (RT-PCR) is a sensitive technique that can detect prostate-specific messenger RNA in circulating blood. Many authors have studied the potential of RT-PCR as a staging technique in prostate cancer (PC). Clinical sensitivity and in some cases specificity has been disappointing. Few authors have been able to correlate RT-PCR result with patient stage. We have compared the results of using two different RT-PCR protocols with different sensitivities on blood samples from prostate cancer patients. An 80-amplification-cycle nested primer RT-PCR assay had a detection limit of 10 prostate cells and a 50-cycle RT-PCR could detect 20 cells in 5 ml blood. The 80-cycle assay detected prostate mRNA in four of 10 female samples, whereas the 50-cycle assay detected it in none. There was little difference in the assays’ ability to detect prostate mRNA in advanced PC patients. The 50-cycle assay could differentiate between hormone-escaped, stable hormone-treated and untreated localized PC patients, whereas the 80-cycle assay could not. Each blood sample must be assayed several times with RT-PCR to avoid false-negative results and, if this is done, assay specificity can be increased with little effect on clinical sensitivity. © 2000 Cancer Research Campaign


*British Journal of Cancer* (2000) **83**(8), 992–997
© 2000 Cancer Research Campaign
doi: 10.1054/ bjoc.2000.1417, available online at http://www.idealibrary.com on IDEAL®

### Optimization of the reverse transcriptase polymerase chain reaction for the detection of circulating prostate cells

**IG McIntyre[1,3], K Spreckley[2], RB Clarke[2], E Anderson[2], NW Clarke[2,3] and NJR George[1]**

[1]Department of Urology, Withington Hospital, Manchester M21 1AH; [2]Clinical Research Department, Christie Hospital, Manchester M20 4BX; [3]Department of Urology, Hope Hospital, Stott Lane, Salford M6 8HD, UK

**Summary** The reverse transcriptase polymerase chain reaction (RT-PCR) is a sensitive technique that can detect prostate-specific messenger RNA in circulating blood. Many authors have studied the potential of RT-PCR as a staging technique in prostate cancer (PC). Clinical sensitivity and in some cases specificity has been disappointing. Few authors have been able to correlate RT-PCR result with patient stage. We have compared the results of using two different RT-PCR protocols with different sensitivities on blood samples from prostate cancer patients. An 80-amplification-cycle nested primer RT-PCR assay had a detection limit of 10 prostate cells and a 50-cycle RT-PCR could detect 20 cells in 5 ml blood. The 80-cycle assay detected prostate mRNA in four of 10 female samples, whereas the 50-cycle assay detected it in none. There was little difference in the assays' ability to detect prostate mRNA in advanced PC patients. The 50-cycle assay could differentiate between hormone-escaped, stable hormone-treated and untreated localized PC patients, whereas the 80-cycle assay could not. Each blood sample must be assayed several times with RT-PCR to avoid false-negative results and, if this is done, assay specificity can be increased with little effect on clinical sensitivity. © 2000 Cancer Research Campaign

**Keywords**: prostate cancer; reverse transcriptase polymerase chain reaction; micrometastasis

The reverse transcriptase polymerase chain reaction (RT-PCR) is a sensitive molecular technique that can be used to detect tumour cells in peripheral blood. RT-PCR has been used in the diagnosis of haematological malignancies for some time (Kawasaki et al, 1988) but it is now being investigated as a technique for detecting early metastases from solid tumours. At current levels of sensitivity RT-PCR should in theory be able to detect a total of 1000 cancer cells in the circulating blood. This should give it a considerable advantage over conventional techniques, which can detect metastatic deposits only once they contain several million tumour cells.

The ability to detect small or micrometastases would be of particular value in the management of prostate cancer (PC) patients being considered for radical prostatectomy, since many of these patients develop prostate-specific antigen (PSA) recurrence after surgery. Several workers have studied the use of RT-PCR with primers specific to PSA and prostate-specific membrane antigen (PSMA) for this purpose. PSA is a serum marker that is widely used in the diagnosis and monitoring of prostate cancer patients. It is almost completely prostate-specific but is not secreted by all prostate tumours and can be unreliable in the monitoring of patients with hormone-escaped PC. PSMA is also prostate-specific but serum PSMA measurements are not yet widely used in the management of PC. It is possible that PSMA may be secreted more reliably than PSA in hormone-escaped disease. RT-PCR for the messenger RNA (mRNA) for both of these prostate-specific proteins should provide a sensitive and specific test for the presence of prostate cells in the circulation.

A number of groups have now reported their results obtained from investigations into the use of RT-PCR for detecting circulating prostate cells. Only one group has been able to show a consistent correlation between the presence of circulating cells detected preoperatively using RT-PCR (with PSA primers) and the pathological failure of radical surgery (Katz et al, 1994). Others have been unable to demonstrate such a correlation (Corey and Corey, 1998). There have been similar problems correlating RT-PCR results with clinical stage in patients with breast or colorectal carcinomas (Raj et al, 1998). These disappointing results may be related to assay sensitivity. Most authors claim to be able to detect one prostate cell diluted in 1 million blood cells with RT-PCR (Corey and Corey, 1998) but the number of advanced prostate cancer patients with detectable circulating prostate cells varies from 25–100% (Corey and Corey, 1998; Grasso et al 1998) depending on the assay used. Furthermore, a number of authors have reported positive results in samples taken from patients who do not have prostate cancer when using the most sensitive RT-PCR techniques (Smith et al, 1995; Henke et al, 1997).

The aim of the present study was to develop a robust nested RT-PCR method with both PSA and PSMA primers and to optimize the assay sensitivity and specificity by titration against a set of blood samples from patients with varying stages of prostate cancer and against a panel of samples in which no prostate cells were present.

*Received 5 January 2000*
*Revised 6 June 2000*
*Accepted 27 June 2000*

*Correspondence to:* IG McIntyre[3]

### PATIENTS AND METHODS

### Patients

Venous blood was collected from patients with prostate cancer during routine follow-up clinic visits. These patients were grouped according to the following criteria:

Group 1: patients with hormone-escaped metastatic prostate cancer with bone pain and/or serum PSA over 100 ng $l^{-1}$ ($n$ = 9)
Group 2: hormone-manipulated patients with serum PSA less than 10 ng $l^{-1}$ and stable ($n$ = 14)
Group 3: untreated patients with clinically localized disease and a PSA less than 50 ($n$ = 19)
Group 4: normal female volunteers ($n$ = 10).

### Methods

A 5 ml blood sample was collected from each of these subjects into 0.02 M EDTA and processed for RNA extraction within 8 h. To extract RNA, the 5 ml whole blood sample was layered onto a 5 ml cushion of Lymphoprep (Nycomed, Oslo, Norway), a 1.077 ng $ml^{-1}$ density medium, and centrifuged at 2000 rpm (700 ***g***) for 20 min. The buffy layer containing tumour cells and lymphocytes was aspirated and washed in sterile phosphate buffered saline. The resulting cells were then suspended in 1 ml Trizol (Life Technologies, Paisley, UK) and stored at –70°C until RNA extraction which was always carried out within 1 month.

For RNA extraction, the cell pellet in Trizol was thawed and allowed to stand at room temperature (RT) for 5 min. Total RNA was extracted with chloroform, washed in ethanol and dissolved at 55°C in sterile water treated with diethylpyrocarbonate (DEPC (Sigma, St. Louis, USA)). One microgram of the extracted RNA was added to a reverse transcription reaction containing 0.5 μl mixed deoxynucleotide triphosphates (each 20 mM; dNTPs), 0.5 μg random primers and 5 μl reverse transcriptase buffer (all supplied by Promega, Southampton, UK) made up to a total volume of 25 μl with DEPC-treated water. After denaturation at 65°C for 5 min, 400 Units of Moloney murine leukemia virus (M-MLV) reverse transcriptase (Promega) was added and the reaction mixture incubated at 42°C for 60 min. The reaction was stopped by heating to 95°C for 5 min and the resulting complementary DNA (cDNA) product stored at –20°C until PCR analysis.

In order to confirm the integrity of the extracted RNA, the reverse transcribed cDNA was amplified using primers (see Table 1) against the housekeeping gene β2-microglobulin. The PCR mixture contained 1 μl cDNA, 10 μl Taq polymerase 10 × buffer (with 15 mM $MgCl_2$), 1 μl mixed dNTPs (20 mM), 1 μl of the relevant primers and 2 Units of Taq polymerase (Promega) adjusted to a total volume of 100 μl with sterile distilled water. Amplification was carried out in a Hybaid Omnigene thermocycler using a protocol of 95°C for 5 min, followed by 40 cycles of 60°C for 30 s, 72°C for 60 s and 95°C for 30 s, culminating in a prolonged chain elongation step of 72°C for 10 min. 30 μl of the resulting PCR product was subjected to electrophoresis in a 1% agarose gel and visualized by staining with ethidium bromide and UV transillumination.

Samples yielding a β2-microglobulin PCR product of the appropriate size were then analysed using primers for PSA and PSMA (see Table 1). The PCR protocol was the same as that used above except that 2 μl of cDNA was used in each reaction and then 2 μl of the resulting PCR product was put into a second 'nested' reaction using the primers detailed in Table 1. Each cDNA sample was assayed in duplicate using primers for both PSA and PSMA.

Fidelity of amplification was established for the nested primer products by sequencing using the ABA 373 sequencer and was found to be accurate to within one base pair of the expected sequences for PSA and PSMA.

The sensitivity of the RT-PCR protocol was determined by assaying samples of volunteer blood to which limiting dilutions of LNCaP prostate cancer cells were added. Patient samples were then assayed in batches of eight, each containing a negative control to which water rather than cDNA had been added and a positive control consisting of cDNA from LNCaP cells. If a positive result was obtained for the negative control or the positive control was negative, the PCR was repeated.

### RESULTS

### Determination of RT-PCR sensitivity

The initial experiments on LNCaP cultured prostate cancer cells diluted in blood showed that RT-PCR using only the outside primers for either PSA or PSMA and 40 cycles of amplification was sufficient to detect 1000 cells 5 $ml^{-1}$ in all cases and 100 cells 5 $ml^{-1}$ in some cases. Addition of a further 40 PCR cycles using the nested primers increased sensitivity such that 100 cells 5 $ml^{-1}$ could be detected always, 10 cells 5 $ml^{-1}$ frequently (80% of times) and 1 cell 5 $ml^{-1}$ occasionally (10% of times) (Figure 1). However,

**Table 1** Details of outer and nested primers used to amplify β2-microglobulin, PSA and PSMA cDNA

| cDNA | Product size | Forward primer<br>Reverse primer |
|---|---|---|
| β2 microglobulin | 200 bp | 5′-CAT CCA GCG TAC TCC AAA GA-3′<br>5′-GAC AAG TCT GAA TGC TCC AC-3′ |
| PSA outer<br>(Katz et al 1994) | 825 bp | 5′-GAT GAC TCC AGC CAC GAC CT-3′<br>5′-CAC AGA CAC CCC ATC CTA TC-3′ |
| PSA nested | 400 bp | 5′-CAT TGA ACC AGA GGA GTT CTT G-3′<br>5′-CCT CAC ACC TAA GGA CAA AGG-3′ |
| PSMA outer<br>(Israel et al, 1994) | 647 bp | 5′-ATG TCA TTC TGG GAG GTC-3′<br>5′-ACA CCA TCC CTC CTC GAA CC-3′ |
| PSMA nested | 234 bp | 5′-CCT AAC AAA AGA GCT GAA AAG C-3′<br>5′-ACT GTG ATA CAG TGG ATA GCC-3′ |

All primers from GibcoBRL, ••• (author to supply)

© 2000 Cancer Research Campaign 

**Table 2** Minimum number of cultured LNCaP cells 5 ml$^{-1}$ blood reliably detected (i.e. more than 80% of the time) using different nested RT-PCR protocols

| Outside primers (cycles) | Nested primers (cycles) | PSA RT-PCR (LNCaP cells 5 ml$^{-1}$ blood) | PSMA RT-PCR (LNCap cells 5 ml$^{-1}$ blood) |
|---|---|---|---|
| 40 | 40 | 10 | 10 |
| 20 | 30 | 100 | 50 |
| 25 | 25 | 20 | 20 |
| 20 | 20 | 1000 | 1000 |

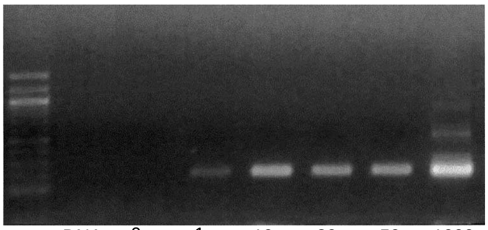

**Figure 1** Gel photograph showing the result of a nested PSMA RT-PCR assay with a total of 80 amplification cycles in detecting different numbers of LNCaP cells added to 5 ml blood samples. The assay in this case was able to detect one LNCaP cell added to the blood

**Table 3** Results obtained when patient and normal volunteer samples were analysed using a '40 plus 40' cycle nested RT-PCR protocol. All samples were assayed in duplicate using primers against PSA and PSMA on two separate occasions and were regarded as positive even if the presence of PSA or PSMA mRNA could be demonstrated once only

| Patient group | *n* | RT-PCR positive (%) |
|---|---|---|
| Hormone-escaped | 9 | 7 (78) |
| Hormone-manipulated | 15 | 12 (80) |
| Untreated | 19 | 14 (74) |
| Normal female control | 10 | 4 (40) |

**Table 4** Results obtained when patient and normal volunteer samples were analysed using a '25 plus 25' cycle nested RT-PCR protocol. All samples were assayed in duplicate using primers against PSA and PSMA on two separate occasions and were regarded as positive even if the presence of PSA or PSMA mRNA could be demonstrated once only

| Patient group | *n* | RT-PCR positive (%) |
|---|---|---|
| Hormone-escaped | 9 | 6 (67) |
| Hormone-manipulated | 15 | 1 (7) |
| Untreated | 19 | 5 (26) |
| Normal female control | 10 | 0 (0) |

differences in the intensities of duplicate bands were a consistent finding in this study, especially when small numbers of LNCaP cells were being analysed. This observation did not appear to be due to experimental error and is discussed in more detail below.

The effects of altering the number of PCR cycles on assay sensitivity is shown in Table 2, where the detection limit is defined as the smallest number of LNCaP cells 5 ml$^{-1}$ blood detectable over

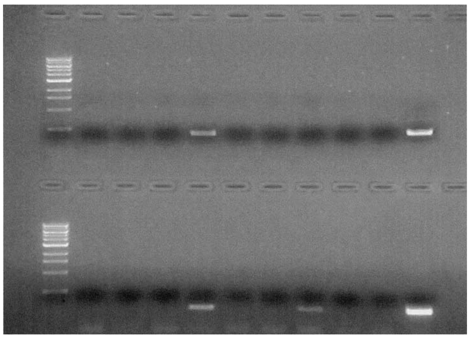

**Figure 2** Gel photograph showing the results of simultaneous nested RT-PCR assays with a total of 50 amplification cycles and PSA primers (top) and PSMA primers (bottom). The numbers along the bottom of the gel refer to patient samples. Patient 3 had advanced prostate cancer and is positive with both PSA and PSMA primers. Patients 2, 4, 7 and 8 have stable hormone-treated disease and patients 1, 5 and 6 are watchful-waiting patients. Of the watchful-waiting patients, patient 5 is positive for the PSMA but not PSA RT-PCR but patients 1 and 6 are negative four assays. The '40 plus 40' cycle RT-PCR protocol was the most sensitive as it reliably detected 10 cells 5 ml$^{-1}$ blood using primers for both PSA and PSMA.

### RT-PCR of patient samples

The '40 plus 40' cycle nested PCR protocol was then used to examine blood samples from the 43 prostate cancer patients described above and from 10 normal female volunteers. The PSA and PSMA mRNA content of each sample was assessed in duplicate in two separate assays and samples were regarded as being positive even if the presence of PSA or PSMA mRNA could be demonstrated once only. The results presented in Table 3 show that the specificity of the '40 plus 40' protocol was poor, as four of the 10 samples from normal females apparently contained PSA or PSMA mRNA. Furthermore, the assay was unable to differentiate between hormone-manipulated patients who were expected to have low numbers of circulating prostate cancer cells (80% positive) and untreated patients who were expected to have higher numbers (74% positive). Therefore, in an attempt to increase assay specificity, the same patient samples were re-analysed using the '25 plus 25' cycle nested PCR technique which was able to detect 20 prostate cells (Table 2). Patient results using this assay are presented in Table 4 and shown in Figure 2. None of the samples taken from normal females yielded a positive result and it was possible to distinguish patients with hormone-treated disease (7% positive) from those with untreated disease (28%

*© 2000 Cancer Research Campaign*

positive) ($\chi^2 < 0.001$ by 2-test). There was little effect on the proportion of positive results obtained from patients with escaped disease (67% vs 78% in the two assays).

Further attempts at increasing assay sensitivity by doubling the amount of RNA used in the reverse transcription reaction resulted in loss of specificity, as judged by the re-appearance of false-positive results in the samples from normal females (data not shown).

### DISCUSSION

Accurate staging continues to be a problem in the management of prostate cancer. It has been suggested that the presence of prostate cancer cells in the peripheral circulation is indicative of occult micrometastases. To this end, several groups have developed sensitive RT-PCR methods that can detect prostate-specific mRNA, but the ability of these assays to predict failure of radical prostate surgery has been disappointing. We believe that this lack of discriminatory power could be due to the enhanced sensitivity of most RT-PCR protocols being achieved at the expense of specificity. We used an RT-PCR assay to detect the mRNA of two different prostate-specific genes, titrating the number of cycles of amplification against a set of samples from patients with varying stages of prostate cancer and against samples in which no prostate cells were present. After doing this we found that a ‘25 plus 25’ cycle nested RT-PCR protocol, while less sensitive than protocols involving more cycles, was more specific and could distinguish advanced prostate cancer patients from those with minimal or hormone-controlled disease. In the process, we encountered a number of problems associated with high-sensitivity RT-PCR that may have contributed to the disappointing results obtained previously, and whose solution may be of interest to other groups.

The first problem encountered was that of false-positive results. Using the ‘40 plus 40’ cycle protocol either PSA or PSMA mRNA were detected in four of the 10 samples from normal females and 11 of the 15 samples from patients known to have stable hormone-manipulated disease. Other groups have reported positive RT-PCR results in samples expected to have few or no prostate cells (O’Hara et al 1996; Smith et al 1995; Henke et al, 1997). Most of these groups have used nested primer RT-PCR assays with a total of 80 PCR cycles. There are a number of potential causes of false positive results.

First, the samples may have become contaminated at some stage in the protocol. We considered this to be unlikely as the negative controls (–cDNA) included in all our assays were invariably negative.

The second possibility is that low levels of PSA and PSMA mRNA are present in female blood samples. It has recently been shown that trace amounts of both PSA and PSMA are detectable in organs other than the prostate (breast in the case of PSA (Yu et al, 1994) and kidney in the case of PSMA (Dumas et al, 1999)). Release of occasional cells from these organs into the bloodstream may be enough to produce a positive RT-PCR result. Indeed Lehrer et al (1996) showed that many blood samples from breast cancer patients were positive for PSA RT-PCR assays.

Thirdly, it has been shown that prostate-derived RNA can be detected in blood leucocytes, presumably after phagocytosis of circulating prostate epithelial cells (Lintula and Stenman, 1997).

The final and most likely reason behind high rates of false-positivity is that the most sensitive RT-PCR assays can detect the small amounts of mRNA derived from illegitimate gene transcription (Chelly et al, 1989). Because of leaky transcription control one mRNA molecule for every gene in the genome, including PSA and PSMA, is likely to be present in a sample of approximately $10^5$ white blood cells. Nested RT-PCR protocols involving a total of 80 amplification cycles may be sensitive enough to detect these occasional molecules, especially when the RNA under study has been extracted from samples with large numbers of white blood cells. Recent studies using RT-PCR primers for non-prostatic epithelial tumours have shown high rates of positive RT-PCR results for patients with non-neoplastic chronic inflammatory conditions (Jung et al, 1998). This is thought be due to cytokines increasing the level of illegitimate transcription of the mRNA under study.

By reducing the sensitivity of the RT-PCR assay we reduced the likelihood of detecting illegitimate transcripts. The in vitro sensitivity of the assay was slightly reduced from a detection threshold of 10 LNCaP cells in 5 ml blood to a threshold of 20 cells. However, there was little reduction in the sensitivity of the assay in detecting circulating prostate cells in patients with hormone-escaped disease. Significantly, the specificity of the assay was increased such that positive results were obtained in none of the female patients and only one of the hormone-controlled PC patients. We feel that this reduced in vitro assay sensitivity is more than compensated for by high specificity.

Another way of reducing the possibility of illegitimate transcription would be to carry out immunomagnetic depletion of the white blood cells or enrichment of the prostate cells in the blood sample. This technique has been used successfully with a limited series of prostate cancer patients (Makarovskiy et al, 1997). However, we have found that such techniques can result in loss of up to half of the prostate cells in the blood sample (data not shown) and they add greatly to experimental complexity.

The second problem encountered in the present study was one of poor duplication. For example when using the ‘25 plus 25’ cycle RT-PCR assay to detect PSA mRNA in duplicate, one replicate would often give a positive result and the other a negative one. We initially ascribed this poor duplication to experimental error but it became clear that this was not the case and the problems with the duplicates could be put down to the probability of sampling PSA or PSMA cDNA when removing aliquots of solutions containing extremely dilute cDNA solutions.

A single prostate cell is likely to contain approximately 300 PSA or PSMA mRNA molecules and thus 10 prostate cells (our first assay’s sensitivity limit) will contain 3000 such mRNA molecules. The RT-PCR assay described uses 1% of the total RNA extracted from blood samples corresponding to an average of 30 mRNA molecules in this case. These 30 molecules are converted to cDNA and transferred to the PCR reaction by micropipette. Even if the pipette is extremely accurate, there will be variation in the number of molecules of cDNA added to the PCR reaction because of sampling error.

As already discussed, an oversensitive PCR reaction will give false-positive results because of illegitimate transcription. Assuming a white blood cell (wbc) count of 5 million $ml^{-1}$ and one illegitimately transcribed prostate RNA molecule per 100 000 wbc there will be 250 illegitimately transcribed PSA or PSMA mRNA molecules in a 5 ml blood sample. The PCR sensitivity should not be so high as to detect these molecules. The only accurate way of calibrating the PCR sensitivity is to run the RT-PCR reaction against a number of female blood samples. If *any* are found to be positive, then the PCR sensitivity must be reduced.

© 2000 Cancer Research Campaign

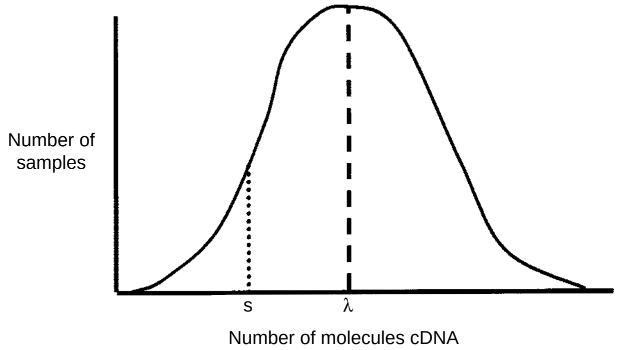

**Figure 3** Graph showing the distribution of probability of sampling a certain number of molecules of cDNA from an extremely dilute solution. λ is the mean number of molecules in the solution and S is the number of molecules required to give a positive RT-PCR result (i.e. the RT-PCR sensitivity). The probability of a false-negative result is the proportion of the area under the curve to the left of S

Once the sensitivity of the RT-PCR assay has been set in this way then the probability of each individual PCR reaction giving a positive result with a sample at near the desired threshold of assay sensitivity (for example 10 LNCaP cells in 5 ml blood) can be estimated with the Poisson distribution.

Figure 3 shows a Poisson distribution curve with the number of molecules of cDNA per sample of a given dilute cDNA solution on the horizontal axis and the likelihood of that number of molecules being in the sample on the vertical axis. The most likely number of molecules, λ, would be 30 in the above case of a blood sample with 10 prostate cells in it. There is, however, a small chance of having a very small or very large number of molecules in the sample and this is represented by the two ends of the Poisson curve. The sensitivity limit of the PCR, the smallest number of cDNA molecules which will give a positive result, is represented on the graph by the point S. The probability of a positive result is the area under the curve to the right of S, divided by the total area under the curve. If there are a large number of prostate cells in the blood sample then the value of λ will be high and the probability of a positive PCR result will be high. This probability has been found by one worker (Jung et al, 1997) to be in the order of 60% for a number of prostate cells at the sensitivity limit of a typical RT-PCR assay (10 cells in 5 ml blood in our case). The number of repeat PCR reactions needed to ensure a positive result can be calculated from a Poisson table. This shows that a sample needs to be RT-PCR assayed five times to ensure 98% probability of a correct result, if the probability of one assay being positive is 60%. We found that our '25 plus 25' cycle PCR assay could detect 20 LNCaP cells diluted in 5 ml blood consistently, and 10 LNCaP cells in 5 ml blood on approximately 50% of occasions. Thus, five repeat PCR reactions would be needed to be 98% sure of detecting the mRNA in 10 LNCaP cells, whereas only one assay is needed to detect the mRNA in 20 such cells.

After adjusting the assay sensitivity and specificity against control blood samples it should be fine-tuned with samples from patients with known prostate cancer. In this study fine-tuning was demonstrated by the ability of the '25 plus 25' cycle RT-PCR assay to differentiate the hormone-treated from untreated groups ($\chi^2 < 0.001$) whereas the '40 plus 40' cycle RT-PCR could not.

If a specific number of tumour cells are required in the circulation before disease progression is likely to occur, and this number has been suggested to be two prostate cells per ml blood (Fidler, 1990), then the RT-PCR assay should be calibrated accordingly. It should not regularly detect small amounts of prostate RNA in the circulation within leucocytes or prostate cancer cells too small in number to be clinically significant, though such small numbers of cells will occasionally be detected even by relatively insensitive RT-PCR assays. It is also important to assay each sample an adequate number of times to allow for sampling error.

The implications of being unable to assay the complete mRNA complement of an individual blood sample have been discussed. The implications of sampling error in taking a 5 ml blood sample from a total circulating volume of 5 L are similar, especially when the cells under study are very sparse. This further increases the likelihood of a false-negative RT-PCR result in a patient with a small number of circulating prostate cells.

This study has shown that highly sensitive RT-PCR assays can detect very small numbers of circulating prostate cells, but this is at the expense of assay specificity. Lowering the sensitivity of the assay not only improves its specificity, but also improves its ability to differentiate between different groups of prostate cancer patients. For statistical reasons it is important to subject each sample to repeated assays when using less sensitive RT-PCR protocols. However, because of the cumulative sampling errors of taking a small sample of the patient's total circulating blood and only amplifying a small proportion of this in the RT-PCR assay, it is inevitable that false-negative results will occur in a small proportion of patients.

### ACKNOWLEDGEMENTS

The authors thank the British Urological Foundation for their support for this project and Dr Graham Turnock for mathematical advice.

### REFERENCES

Chelly J, Concordet J-P, Kaplan J-C and Kahn A (1989) Illegitimate transcription: transcription of any gene in any cell type. *Proc Natl Acad Sci USA* **86**: 2617–2621

Corey E and Corey MJ (1998) Detection of disseminated prostate cells by reverse transcription polymerase chain reaction (RT-PCR): technical and clinical aspects. *Int J Cancer* **77**: 655–673

Dumas F, Gala JL, Brasseur F, Eschwege P, Paradis V, Lacour B, Philippe M and Loric S (1999) Molecular expression of PSMA mRNA and protein in renal tumours. *Int J Cancer* **80**: 799–803

Fidler IJ (1990) Critical factors in the biology of human cancer metastasis: twenty eighth GHA Clowes Memorial Lecture. *Cancer Res* **50**: 6130–6138

Grasso YZ, Gupta MK, Levin HS, Zippe CD and Klein EA (1998) Combined nested RT-PCR assay for prostate specific antigen and prostate specific membrane antigen in prostate cancer patients: correlation with pathological stage. *Cancer Res* **58**: 1456–1459

Henke W, Jung M, Jung K, Lein M, Schlechte H, Berndt C, Rudolph B, Schnorr D and Loening SA (1997) Increased analytical sensitivity of RTPCR of PSA mRNA decreases diagnostic specificity of detection of prostate cells in blood. *Int J Cancer* **70**: 52–56

Israeli RS, Miller WH Jr, Su SI, Powell T, Fair WR, Samadi DS, Huryk RF, DeBlasio A, Edwards ET, Wise GJ and Heston WDW (1994) Sensitive nested reverse transcriptase polymerase chain reaction detection of circulating prostatic tumour cells: comparison of prostate-specific membrane antigen and prostate-specific antigen based assays. *Cancer Res* **54**: 6306–6310

Jung R, Ahmad-Nejad P, Wimmer M, Gerhard M, Wagener C and Neumaier M (1997) Quality management and influential factors for the detection of single

 © 2000 Cancer Research Campaign cells by reverse transcriptase polymerase chain reaction. *Eur J Chem Clin Biochem* **35**: 3–19

Jung R, Kruger W, Hosch S, Holweg M, Gutensohn K, Wagener C, Neumaier M and Zander AR (1998) Specificity of reverse transcriptase polymerase chain reaction assays designed for the detection of circulating cancer cells is influenced by cytokines in vivo and in vitro. *Br J Cancer* **78**: 1194–1198

Katz AE, Olsson CA, Raffo AJ, Cama C, Perlman H, Seaman E, O'Toole KM, McMahon D, Benson MC and Buttyan R (1994) Molecular staging of prostate cancer with the use of an enhanced reverse transcriptase PCR assay. *Urology* **43**: 765–775

Kawasaki ES, Clark SS, Coyne MY, Smith SD, Champlin R and Wittle ON (1988) Diagnosis of chronic myeloid leukemia and acute lymphocytic leukemias by detection of leukemia-specific mRNA sequences amplified in vitro. *Proc Natl Acad Sci USA* **85**: 5698–5702

Lehrer S, Terk M, Piccoli SP, Song HK, Lavagnini P and Luderer AA (1996) Reverse transcriptase-polymerase chain reaction for prostate-specific antigen may be a prognostic indicator in breast cancer. *Br J Cancer* **74**: 871–873

Lintula S and Stenman UH (1997) The expression of prostate-specific membrane antigen in peripheral blood leucocytes. *J Urol* **157**: 1969–1972

Makarovskiy AN, Ackerley III W, Wojcik L, Halpert G, Stein BS, Carreiro MP and Hixson DC (1997) Application of immunomagnetic beads in combination with RTPCR for the detection of circulating prostate cancer cells. *J Clin Lab Analysis* **11**: 346–350

O'Hara SM, Veltri RW and Skirpstunas P (1996) Basal PSA mRNA levels detected by quantitative reverse transcriptase polymerase chain reaction in blood from patients without prostate cancer. *J Urol* **155(Suppl)**: 430A

Raj GV, Moreno JG and Gomella LG (1998) Utilisation of polymerase chain reaction technology in the detection of solid tumours. *Cancer* **82**: 1419–1442

Smith MR, Biggar S and Hussain M (1995) Prostate-specific antigen messenger RNA is expressed in non-prostatic cells: implications for detection of micrometastases. *Cancer Res* **55**: 2640–2644

Yu H, Diamendis EP and Sutherland DJ (1994) Immunoreactive prostate-specific antigen levels in female and male breast tumours and its association with steroid hormone receptors and patient age. *Clin Biochem* **27**: 75–79

*© 2000 Cancer Research Campaign*
